# Midgut Epithelial Dynamics Are Central to Mosquitoes’ Physiology and Fitness, and to the Transmission of Vector-Borne Disease

**DOI:** 10.3389/fcimb.2021.653156

**Published:** 2021-03-25

**Authors:** Bretta Hixson, Mabel Laline Taracena, Nicolas Buchon

**Affiliations:** Department of Entomology. Cornell Institute of Host-Microbe Interactions and Disease, College of Agriculture and Life Sciences, Cornell University, Ithaca, NY, United States

**Keywords:** *Aedes*, *Anopheles*, mosquito, vector, intestinal stem cell, midgut epithelium, tolerance, resistance

## Introduction

Hematophagous mosquitoes vector many important human diseases, and a detailed understanding of their physiology is crucial for designing efficient vector-control strategies. The midgut epithelium plays a central role in mosquito physiology: as a digestive tissue, it transitions between processing diets of sugar and blood to support both nutrition and reproduction; as a niche for microbiota, it balances immunity and tolerance to maintain a functional microbiome; and in its capacity as an interface between the mosquito and human pathogens, it serves as a barrier that often bottlenecks parasites as they travel from gut lumen to hemocoel, *en route* to the salivary glands and their next host (Smith et al., 2014; Franz et al., 2015).

Studies in model organisms, such as *Drosophila*, have revealed that the insect gut epithelium can be highly complex and dynamic. The *Drosophila* midgut epithelium comprises diverse cell types, including polyploid enterocytes (ECs), enteroendocrine cells (EEs), and undifferentiated progenitors (intestinal stem cells, ISCs, and enteroblasts, EBs) (Bonfini et al., 2016). These cells communicate and coordinate to maintain the gut’s function and integrity. *Drosophila* ISCs respond to diverse stimuli with symmetric and asymmetric divisions to continuously replenish the midgut epithelium or plastically alter its structure (O’Brien et al., 2011). Inputs including nutrition (O’Brien et al., 2011), endocrine signaling (Reiff et al., 2015; Ahmed et al., 2020), the presence/composition of the microbial community (Buchon et al., 2009a), pathogenic challenge (Buchon et al., 2009b; Houtz et al., 2017), reactive oxygen species (Hochmuth et al., 2011), and aging (Biteau et al., 2008) dynamically alter the kinetics of division, differentiation, and endocycling to reshape epithelial composition.

In contrast to *Drosophila*, the epithelial composition and dynamics of mosquito midguts are little studied. Early histological studies noted EEs in the mosquito midgut epithelium (Hecker, 1977; Brown et al., 1985), and mapped their production of neuropeptides (Veenstra et al., 1995), but their physiological significance remains unexplored. Likewise, the presence of putative “regenerative cells” in the adult midgut epithelium was noted, but they were believed to be mitotically inactive (Billingsley, 1990). However, a growing number of publications have documented DNA synthesis and/or mitoses in the midguts of several mosquito species (Baton and Ranford-Cartwright, 2007; Hernández-Martínez et al., 2013; Janeh et al., 2017; Serrato-Salas et al., 2018; Taracena et al., 2018; Janeh et al., 2019; Maya-Maldonado et al., 2020), indicating that the mosquito midgut epithelium is also highly responsive and dynamic. Furthermore, a recent study using single-cell RNA-sequencing of the midgut of *Aedes aegypti* revealed multiple cell clusters that express markers corresponding to all major cell types described in the *Drosophila* midgut (Cui and Franz, 2020). Here, we discuss several mechanisms by which we anticipate that mosquito midgut epithelial dynamics may influence the parameters of fitness, vector competence, and vectorial capacity. We propose that midgut epithelial dynamics are an important and underexplored frontier in the study of mosquito physiology.

## Epithelial Dynamics in Gut Maturation, Blood-Feeding, Aging, and Interactions with the Gut Microbiota

The mosquito midgut begins in the larval stage as a lattice of diploid regenerative cells and larger endoreplicating cells (Ray et al., 2009). During the larval to pupal molt, all polyploid cells are lost, leaving only a network of diploid cells (Nishiura et al., 2003). This pool of cells is likely homologous to the adult midgut progenitors in *Drosophila*, which give rise to the adult midgut in the final stage of development (Hartenstein et al., 1992; Houtz et al., 2019). In *Anopheles albimanus* mosquitoes, it was observed that the adult midgut continues to mature in the 24 hours following emergence, as proportions of diploid cells fall, and polyploid cells (4N and 8N) accumulate *via* endocycling (Maya-Maldonado et al., 2020). This post-emergence maturation phase is marked in mosquitoes by elevated titers of juvenile hormone (JH) (Zhu and Noriega, 2016). Within a few days of emergence, the adult female is ready to take her first blood-meal, stimulating the production of 20 hydroxyecdysone (20E), which circulates in the hemolymph and activates vitellogenesis in the fat body (Martín et al., 2001; Wang et al., 2002; Bai et al., 2010). In *Drosophila*, both JH and 20E are induced by mating, and both promote epithelial proliferation to drive gut growth (Reiff et al., 2015; Ahmed et al., 2020). Loss of either signal compromises fecundity, suggesting that the gut’s growth response is adaptive for optimizing nutrient acquisition to maximize reproductive output. In the mosquito midgut epithelium, there is some evidence that blood-feeding stimulates compositional changes. In the *Aedes albopictus* midgut, blood-feeding induces phosphorylation of ERK, a kinase in the EGFR pathway (Liu et al., 2020) which, in *Drosophila* ISCs, is sufficient to drive proliferation (Buchon et al., 2010). We propose that the pro-proliferative response to JH and 20E may be conserved in the midguts of mosquito species, helping to prepare the epithelium for the task of digesting blood.

Upon acquisition of a blood-meal, the mosquito midgut must pivot to the exploitation of its new diet. A recent single-cell RNA-seq study of *Ae. aegypti* midguts, before and after a blood meal, demonstrated that blood-feeding stimulates an increase in the proportion of putative ISCs/EBs as well as ECs and “EC-like” cells, providing further evidence for a proliferative response (Cui and Franz, 2020). The authors also observed multiple distinct populations of ECs and found that the proportions of EC populations changed in response to blood-feeding. Together, these results suggest that blood-feeding prompts not only the proliferation of progenitors, but changes among differentiated ECs. We propose that these changes could reflect a form of terminal differentiation for immature ECs, which may be key to the digestion of blood.

Midgut epithelial dynamics play an important role in every stage of the insect lifecycle, including its terminus. In *Drosophila*, the aging of the gut epithelium, with associated dysplasia and loss of barrier integrity, is believed to be an important intrinsic limiting factor in the lifespan of the insect (Biteau et al., 2008; Rera et al., 2012). This aging is accelerated by the presence of the microbiota, which increases the rate of epithelial turnover (Buchon et al., 2009a), and pathogenic microbes can drive even more rapid turnover of the gut epithelium (Buchon et al., 2009b). Considering that the female mosquito midgut is subjected not only to all the ordinary stresses of aging but also to the rigors of blood-feeding - periods of intense mechanical strain accompanied by the rapid proliferation of gut microbes – we posit that the midgut may set the limits of mosquito longevity and, by extension, vectorial capacity.

## Epithelial Dynamics and Control Of Infection

A mosquito’s competence as a vector depends on the successful invasion and traversal of midgut epithelial cells by orally acquired pathogens. It has already been shown that epithelial dynamics, in the form of cell sacrifice, participate in the bottlenecking of invading pathogens in mosquito midguts. In the case of *Plasmodium* infection, this phenomenon has been likened to a “time bomb” where the invasion of an epithelial cell by an ookinete commences a countdown culminating in the death and extrusion of the invaded cell (Han et al., 2000). Ookinetes that fail to make their way to the safety of the basal lamina before this extrusion occurs are denied advancement to the oocyst stage of development. By sacrificing epithelial cells, the gut may limit or altogether block the progression of plasmodial infection. By a similar principle, the elimination of epithelial cells may also help to limit viral infections in mosquitoes. Several histological studies have associated viral infection of the midgut with epithelial pathology and cell loss (Weaver et al., 1992; Vaidyanathan and Scott, 2006). There is some evidence that this phenomenon is strain-dependent, and correlates negatively with susceptibility to viral infection. Transcriptomic profiling of the midguts of mosquitoes infected with DENV2 found that a refractory strain showed biased representation of transcripts associated with cell death as compared to a susceptible strain (Behura et al., 2011), and a follow-up study found that silencing of pro-apoptotic genes increased the susceptibility of a partially refractory strain (Ocampo et al., 2013). These observations suggest that epithelial cell elimination may underlie the refractoriness exhibited by some strains to infection with incompatible viruses.

Even in the absence of cell sacrifice, we can envision scenarios in which the dynamics of the epithelial response to an incipient infection might alter its outcome. Intensified endocycling might increase immune capacity. Increased proliferation might alter midgut epithelial composition and the density of host factors required for epithelial invasion. Newly differentiated ECs might be either more or less resistant to infection than their older counterparts. One 2018 study correlated population-level DENV susceptibility to the timing of the proliferative response in the *Ae. aegypti* midgut, suggesting that the activity of progenitor cells might influence the outcome of infection (Taracena et al., 2018). We propose that epithelial dynamics may play an important role in the bottlenecking of all kinds of pathogens and helps to limit vector competence in a species and/or strain-dependent manner.

## Epithelial Repair and Infection Tolerance

While cell sacrifice may help to limit infection, the loss of midgut epithelial cells may negatively impact the survival of the mosquito vector. The time bomb model of *Plasmodium* invasion dictates that every invaded epithelial cell is fated to die (Han et al., 2000). As the majority of invading ookinetes never mature into oocysts (Ghosh et al., 2000), and as ookinetes may move laterally through multiple ECs during invasion (Han et al., 2000), oocyst counts - ranging from 1-10 in the field (Gouagna et al., 1998) to hundreds in the laboratory (Usui et al., 2011) - represent an extremely conservative estimate of EC loss. As previously noted, infection with viral and bacterial pathogens can also drive EC loss. For the mosquito gut, these losses could destroy a substantial proportion of the epithelium; moreover, even a mild infection and the extrusion of a handful of ECs could theoretically result in a fatal loss of gut barrier integrity.

Given the damage that *Plasmodium* infection causes to the midgut, it is remarkable that survival effects in *Plasmodium*-infected mosquitoes appear to be minimal (Han et al., 2000). Despite a potentially significant loss of midgut epithelium, mosquitoes are apparently able to maintain barrier integrity and gut function sufficient to their needs. Mechanisms have been articulated for closing emerging holes in the gut at the moment of EC loss by drawing together neighboring ECs (Han et al., 2000; Gupta et al., 2005), but it is unlikely that this process fully compensates for cell loss. While the midgut may possess ECs significantly in excess of what it needs to maintain adequate function for survival, it is also possible that mosquitoes’ tolerance of midgut damage is dependent on progenitor-mediated replacement of lost epithelial cells. Multiple studies support a homeostatic proliferative role for epithelial progenitors in mosquito vector species. A histological study positively correlated instances of apparent mitosis in the midgut with the intensity of *Plasmodium* infection in *Anopheles stephensi* (Baton and Ranford-Cartwright, 2007)*. Ae. albopictus* and *Culex pipiens* mosquitoes exhibit increased mitoses following oral bacterial infection and chemical challenge (Janeh et al., 2019). Epithelial repair may also be important in response to cell loss sustained during viral infection in non-permissive strains. Even in cases where it does not induce cell loss, viral infection of midgut cells may cause stress and prompt cell signaling responses (e.g. JAK-STAT activation (Behura et al., 2011)) which could stimulate the activity of progenitors. In summary, we propose that the ability to effect proliferative repair may be important for preserving midgut barrier integrity, with potential knock-on effects for mosquito survival and, hence, vectorial capacity.

## Discussion

**Figure 1:**
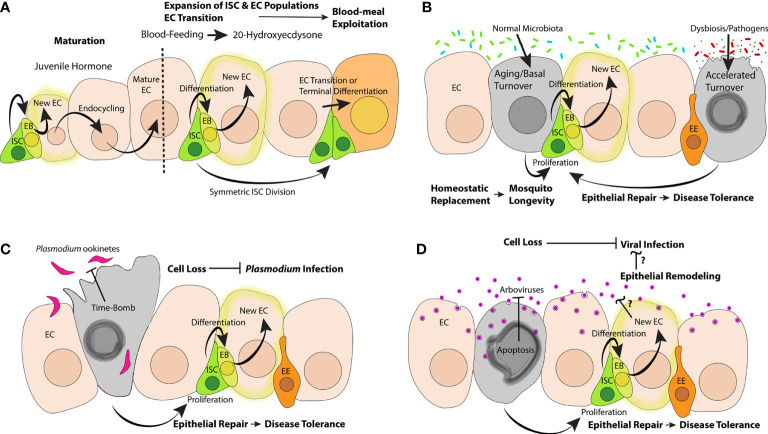
Possible impacts of epithelial dynamics in the mosquito midgut on the hematophagous lifecycle, aging, interactions with gut flora, *Plasmodium* and arboviral infections. **(A)** During the post-emergence maturation, JH could stimulate ISCs to proliferate and create new ECs or prompt ECs to endocycle to attain higher ploidy; blood-feeding stimulates the production of 20E, which could stimulate the proliferation of ISCs, the differentiation of new ECs, and transcriptional changes in ECs. **(B)** Normal microbiota could contribute to aging and basal turnover of EC populations; dying ECs could stimulate ISCs to effect homeostatic replacement; dysbiosis and/or infection with oral bacterial pathogens could accelerate the turnover of epithelial cells; ISC-mediated repair could serve as a disease tolerance mechanism, promoting mosquito survival. Invasion by *Plasmodium* **(C)** and/or arboviral pathogens **(D)** could prompt cell sacrifice mechanisms to limit pathogenic success; ISC proliferation and differentiation could help infected mosquitoes to tolerate epithelial damage.

The successful transmission of mosquito-borne pathogens depends on (a) the maintenance of a sufficiently large and fit vector population (b) the ability of pathogens to progress from oral to systemic infection (essential for vector competence) and (c) the longevity of infected mosquitoes (a key determinant of vectorial capacity). We propose that midgut epithelial dynamics (cell loss, proliferation, differentiation, and endocycling, **Figure 1**) may play an important role in determining the rate of pathogen transmission by: (a) adapting epithelial composition according to hormonal and/or nutritional cues to optimize the exploitation of blood meals, thereby maximizing fecundity (b) setting the natural limits of mosquito lifespan (c) suppressing and killing pathogens as they traverse the midgut barrier and (d) promoting the survival of infected mosquitoes *via* repair mechanisms which help the mosquito to tolerate pathogen-mediated damage. Understanding these dynamics in mosquitos may allow us to develop interventions that will suppress mosquito fecundity, raise barriers to systemic infection, and/or abbreviate the survival of infected mosquitoes.

## Author Contributions

All authors contributed to the article and approved the submitted version.

## Funding

NIH R01AI148529, NIH R21AG065733, NIH R01AI148541 and NSF IOS1656118.

## Conflict of Interest

The authors declare that the research was conducted in the absence of any commercial or financial relationships that could be construed as a potential conflict of interest.
